# Dissolving the Puzzle of Resultant Moral Luck

**DOI:** 10.1007/s13164-015-0249-0

**Published:** 2015-04-01

**Authors:** Neil Levy

**Affiliations:** Florey Institute of Neuroscience and Mental Health, University of Melbourne, Parkville, 3010 Australia

## Abstract

The puzzle of resultant moral luck arises when we are disposed to think that an agent who caused a harm deserves to be blamed more than an otherwise identical agent who did not. One popular (but controversial) perspective on resultant moral luck explains our dispositions to produce different judgments with regard to the agents who feature in these cases as a product not of what they genuinely deserve but of our epistemic situation. On this account, there is no genuine resultant moral luck; there is only luck in what evidence becomes available to observers. In this paper, I develop an evolutionary account of our inclination to take the results of actions as evidence for the mental states of agents, thereby explaining why the resulting intuitions are recalcitrant to correction. The account explains why the puzzle of resultant moral luck arises: because our disposition to take the harms agents cause as evidence of their mental states can produce intuitions which conflict with those that arise when we examine agents’ mental states without reference to the results of their actions. The account also helps to solve the puzzle of resultant moral luck, by providing a strong reason to ignore the intuitions caused by our disposition to regard actual harms as evidence of mental states. Since these intuitions arise using an unreliable proxy for agents’ mental states, they ought to be trumped by more reliable evidence.

Cases involving what Nagel (1979) called resultant moral luck seem to pull us in two contrary directions at the same time. These cases dispose us to blame one agent more than another, but also have features that lead us to think that the agents cannot differ in how much blame they deserve. This gives rise to what I will call the puzzle of resultant moral luck. The puzzle is best illustrated by comparing pairs of cases, in which similarly situated agents perform similar actions, but cause outcomes of widely differing moral significance. Consider, for example, two agents who drive home despite knowing that they have had too much to drink. Both unexpectedly encounter a patch of ice, and in their impaired state lose control of the car; both end up sliding to a halt against a tree. Agent one causes no serious damage, but agent two hits a child playing in the snow and injures her. Both agents seem due some blame, but agent two seems due significantly more blame than agent one, though he took no more or less care than agent one and drove no better or worse.

When we focus on what is common to both cases, especially the equal recklessness or negligence of the agents, we may have the powerful intuition that they must be equally blameworthy. Perhaps both are due a lot of blame, because they acted so badly or because both should have foreseen that tragedy might result; perhaps neither is due much blame, because the harmful consequence was (we may suppose) relatively unlikely: in any case, whatever the appropriate level of blame, it seems to be identical across the agents. But when we focus on the divergent consequences of their acts, we are instead disposed to blame agent two rather more than agent one. Neither option seems fully satisfactory, because each leaves a powerful intuition unaccounted for.^1^

According to what Schinkel (2009) calls epistemic reductionists, the puzzle of resultant moral luck arises because we (rightly or wrongly) take the results of actions as a guide to facts about the agents who cause them: their moral character or their intentions, for instance (Richards 1986; Thomson 1989). Epistemic reductionism faces a standard objection: if our conflicting judgments are the product of our imperfect epistemic situation, then an improvement in our epistemic situation should reduce the conflict we experience: at the limit – when all the relevant facts are in – we should experience no conflict at all. But no matter how completely the details of moral luck cases are filled in, we continue to experience conflicting intuitions. In this paper, I will defend epistemic reductionism, with a twist: I will argue that our disposition to generate conflicting judgments when considering resultant moral luck cases has an evolutionary basis. This account predicts and explains the very intuitions that opponents of epistemic reductionism marshal against it. Its ability to explain intuitions, including the intuitions of its opponents, and their recalcitrance establishes it as an account with a strong claim to truth.^2^

Before I begin to argue for my preferred solution to the puzzle, let me clarify the nature of the responses at issue. The puzzle of resultant moral luck concerns *blame*; specifically, whether and how much we ought to blame an agent who is (causally) responsible for a significant harm.^3^ Theorists of moral responsibility and blame disagree as to the nature and content of blame and closely related judgments (such as moral responsibility judgments). In what follows I will stipulate the sense of ‘blame’ I have in mind. It is a sense that is clearly central to debates in criminal law, public discussions of crime and punishment, and moral philosophy, even if it is not the only one that is at issue in these debates. Moreover, it is also a sense that is clearly central to the debate over resultant moral luck, even if other senses of ‘blame’ might also be at stake.

To blame someone, in the sense of the word meant here, is to hold that just because they have performed a particular action, it is appropriate to expose them to certain kinds of burdens. ‘Blame’, in this sense, is backwards-looking: responses to agents that are justified on consequentialist grounds do not count as blame on this account. The notion of blame just stipulated leaves open how significant the burdens the blamed may deserve can permissibly be. It may be that nothing more onerous than a mild rebuke or placing on them the burden of being expected to account for their conduct is warranted. The solution to the puzzle of resultant luck offered here entails that the paired agents are due the same amount of blame in *this* sense, despite the fact that they differ in the amount of harm they cause. Other notions of blame may be defensible, and may be untouched by the argument offered here.^4^

It should be noted that the solution to the puzzle of resultant moral luck offered turns on claims about our dispositions to attribute blame to agents, rather than on claims directly concerning their blameworthiness. In common with most philosophers, I assume that our dispositions to blame – our intuitions more generally – are an indispensable input into the process whereby we attempt to come to justified assessments of degrees of blameworthiness. My project is demonstrating that some such inputs are unreliable, thereby showing that judgments that take them into account are, to that extent, unjustified.

## What Do Blame Judgments Track?

In setting out the puzzle of resultant moral luck, I claimed that we were pulled in different directions depending on whether we focused on agents’ mental states or on the harms they actually caused. In this section, I will argue for the claim that actors’ mental states and the harms they cause make independent and dissociable contributions to the blame judgments of ordinary people.

One way to defend the claim that our blame responses track actors’ mental states is by philosophical argument. In recent work, Michael McKenna (2012) has argued that the best explanation of the pattern of excuses, justifications and exemptions we accept turns on our perception of agents’ mental states; specifically the quality of will – roughly, the regard for morality and for the moral status of others – with which they acted. An agent’s quality of will is primarily determined by her *beliefs* concerning the likely consequences of her actions, her *desires* concerning those consequences and her *intentions* (assuming that intentions are states over and above beliefs and desires). Excuses, justifications and exemptions attempt to show that the quality of will attributed to an apparent wrongdoer is better than we had thought. Thus, the excuse that she hit you *because she tripped* shows that her action did not express a disregard for your welfare, while a justification shows that she was actually doing the right thing, and thereby expressed, at worse, a neutral quality of will (perhaps she elbowed you to alert you to some danger). Finally, exemptions show that the agent lacked some capacity needed to express a morally bad quality of will: she was too young to appreciate the nature of her act, for instance.

Another way to defend the claim that blame judgments track actors’ mental states is by reference to psychological research. There is a great deal of evidence for what Cushman (2013: 346) calls the *basic process of assigning blame*. First we identify the agent or agents causally responsible for some harm. We then assess the mental states – beliefs, desires and intentions – of the agent or agents at the time of the action. Cushman (2008) reports a series of experiments that systematically varied the beliefs and desires of hypothetical agents who caused harms. Subjects assessed the blameworthiness of agents who foresaw that a harm would result but did not desire it, agents who desired that a harm would occur but did not foresee it, and agents who both foresaw and desired a harm. This work demonstrates that belief and desire contribute independently and additively in the assessment of blame. Additional work confirmed that judgments of whether and how much the person should be punished very closely track judgments of blame (Cushman 2008); moreover, there is a large body of evidence that people’s punishment judgments are insensitive to considerations of deterrence and incapacitation (Carlsmith and Darley 2008), which suggests that consequentialist considerations do not drive such judgments. This evidence strongly suggests that the sense of ‘blame’ at issue is the one in which we are interested here.

Philosophical and psychological evidence therefore converges in supporting the claim that blame judgments track agents’ mental states. But Cushman (2008; Cushman et al. 2009; Alicke 2000; Lench et al. forthcoming) have demonstrated that our blame judgments are also, and independently, responsive to the consequences of agents’ actions. Thus, holding desires and beliefs constant, subjects blame more, and impose heavier penalties, on agents who cause greater harms than those who cause lesser. In fact, subjects are willing to assign some blame to agents who neither foresaw nor desired a harm they caused.

The evidence that our blame judgments respond to actors’ mental states and, independently, to the harm they cause goes some way toward explaining the puzzle of resultant luck. We now know why these cases generate the intuitions we feel. However, we don’t yet know why we experience *conflict* when we consider pairs of cases. If actors’ mental states and the harms they cause contribute independently to blame judgments, why don’t we simply conclude that the luckier actor deserves less blame than the unluckier, because she caused a less severe harm? Evidence that actual consequences contribute independently to assessments of blame does not explain why we *also* feel a pull toward setting aside actual consequences.

To explain the conflict, we need to show something more than that our blame responses track both agents’ mental states and the harms they cause. We need to show that, and why, we are disposed to accept some principle incompatible with one set of intuitions or the other. I shall argue that we are disposed to accept the principle that agents deserve (only) a burden that is commensurate with their mental states. Because we accept this principle, but we continue to feel the force of the intuition that they sometimes deserve more blame than that principle can account for, we experience the conflict on which the puzzle of resultant moral luck turns.

## The Adaptive Function of Blame

In order to understand why we accept the principle just mentioned yet continue to feel the force of the intuition that is sensitive to harmful consequences, we need to turn to the evolutionary function of blame judgments.

Though the precise nature and mechanisms of blame remain a matter of dispute, it is widely agreed that the function of blame and punishment is to promote cooperation (Boyd et al. 2003; Frey and Rusch 2012; Cushman 2013). Under a wide variety of circumstances, organisms do better – increase their inclusive fitness – by cooperating with conspecifics (and sometimes with members of other species) than by pursuing more solitary strategies. Cooperative behavior increases inclusive fitness either because the conspecific shares a sufficient proportion of the organism’s genes, such that aiding it increases the likelihood that the organism will have copies of its genes represented in the next generation, or because aid is reciprocated directly or indirectly, or because cooperation enables the exploitation of resources that cannot be exploited by individuals alone.

Punishment enters the picture for familiar game theoretical reasons: populations of altruistic organisms are vulnerable to invasion by organisms playing more selfish strategies. Unconditional cooperators will be taken advantage of by selfish organisms, who will be fitter than them: cooperation will therefore die out and selfish strategies go to fixation. Conditional cooperation – cooperation only with cooperators – is required to avoid this kind of invasion. Blame is a response that protects against free riding and the collapse of cooperation. It may achieve its adaptive ends either by changing the behavior of noncooperators or by excluding them from participation in exchanges (Nakao and Machery 2012).

In human groups in the environment of evolutionary adaptiveness, an individual represents a surprisingly significant investment of time and energy: “the resource debt that individuals acquire as children and adolescents is not paid off until around 50″ (Sterelny 2012: 30). Since individuals represent such a significant investment, the loss of their potential contribution to the group is extremely costly.^5^ Further, the individual is likely to be closely related to other group members, so harming them directly affects other members’ inclusive fitness. These costs can be reduced if blame tracks properties of agents that predict that they are bad investments: that they are likely to continue to defect from cooperative arrangements. These properties are agents’ mental states and capacities. Since deliberate defection is a much better predictor of future defection than is accidental, blame should track agents’ desires and their beliefs about the consequences of their actions. Hence, I suggest, our sensitivity to agents’ quality of will.

We now have an explanation of why blame responses track agents’ mental states. We still need to explain why the harms they cause make an *independent* contribution to such responses. I suggest that our disposition to blame for harms independently of mental states is a product of the constraints under which blame responses evolved.

Due to the costs or difficulty of gathering accurate information, evolved responses may sometimes utilize proxies for the state it is adaptive to track. Since the mental states of agents are at least partially internal states, mechanisms that generate blame judgments will be required to track proxies to track mental states. They are likely to utilize the outputs of theory of mind modules, which respond to cues ranging from facial expression to gaze direction to explicit utterances. In addition, though, they are likely to be sensitive to consequences because agents often have incentives to conceal their motives from one another. Because our blame responses track mental states, agents have an incentive to attempt to pass off deliberate defection as accidental. It is for this reason, I suggest, that harm contributes independently to blame responses. Because there is a sufficiently high correlation between causing and intending harm in a range of circumstances, we are disposed to take the causation of harm as a sufficient condition for some blame in those circumstances. One effect of this disposition to take harm as a proxy for mental states is that under some conditions we are disposed to blame independently of, and in excess of any contribution to blame judgments of, actors’ actual mental states.

We are now in a position to solve the puzzle of resultant moral luck. The proposal gives us the resources to explain not only (1) why we have a disposition to blame that tracks actors’ mental states and (2) why we nevertheless attribute a degree of blame greater than would be predicted by (1) alone, in cases where the agent causes a harm more severe than they wanted, intended or foresaw; we can also explain why the attribution of blame is not stable: that is, why we experience conflict in our intuitions. Above I promised I would solve the puzzle by showing that agents are implicitly committed to the principle that blame should be commensurate with agents’ mental states. I now have the resources to make good on this promise.

On the account of blame attribution I just sketched, blame responses that are caused by perceiving that an actor has caused a harm are not *alternatives* to blame responses that track mental states; the former responses are themselves mediated by the attribution of mental states. Recent work provides independent evidence for this claim: Lench et al. (forthcoming) showed that the direct effect of the moral luck beliefs of their subjects on perceptions of blameworthiness was reduced when the intention of agent blamed was included as a mediator in a mediation analysis, suggesting that people who believe that moral luck can make an agent more blameworthy hold that belief because they implicitly think that a worse harm reflects the agent’s intentions.^6^ Because of the difficulties of tracking mental states directly, we take harms as *proxies for* mental states. We do not have two rival implicit theories of conditions sufficient for blame, one mental state-based and one harm-based; we have one implicit theory of sufficient conditions for blame, which is mental state-based.

We can now solve the puzzle of resultant moral luck. In a resultant moral luck case, we are torn because we are implicitly disposed to accept the following claims:

1. Agents deserve blame that is commensurate with the quality of their will;
2. Actions that cause a harm stem from a quality of will that matches the severity of the harm caused;
3. The agent who caused the more severe harm has no worse a quality of will than the agent who caused the less severe.

We are implicitly disposed to accept claim 1 because we are disposed to attribute blame based on the quality of agents’ wills (recall that our disposition to blame based on harms normally supports, rather than conflicts with, this disposition). The evidence that people attribute more blame to those who cause more severe harms, independent of what they know about the actor’s mental states, together with my hypothesis that this blame is mediated by an implicit – and nonconscious – attribution of a commensurate mental state to the agent, entails claim 2. In the philosophical context, we are disposed to accept claim 3 because it is stipulated (outside that context, our disposition to attribute mental states to agents on the basis of the harms they cause restricts the frequency with which we encounter the puzzle, but of course there is sometimes good evidence that someone has caused a harm in excess of anything they desired, foresaw or intended).

We experience conflict in cases in which claims 2 and 3 commit us to attributing different qualities of will to an agent. When three is combined with one, we are disposed to think that the agent deserves no more blame than another who did not cause as serious a harm, but when two is combined with one we are disposed to think that that agent deserves more blame than his counterpart. Since we are implicitly committed to all three premises, we are implicitly committed to both judgments. Hence the conflict.^7^

The resulting account of the intuitions generated by resultant moral luck cases is plainly a variety of epistemic reductionism: it explains the intuitions we experience by reference to facts about us and the manner in which we attribute blame to others, rather than facts about the agents who feature in these cases. Unlike other epistemic accounts of moral luck, however, this account predicts the intuitions that have sometimes been wielded against epistemic reductionism. As Adler (1987) and Schinkel (2009) note, contemplating the fact that had the agent been luckier they would not have caused a particular harm does not in fact cause the intuition that they deserve more blame to dissipate; this fact, they believe, counts against the claim that our judgments are a function of our epistemic situation. The account offered actually predicts that our intuitions will be recalcitrant in just the way Adler and Schinkel each highlight. The kinds of psychological processes that underlie our judgments are, like many others we have as a consequence of our evolutionary history, likely to be encapsulated in the sense Fodor (1983) made famous: that is, insensitive to personal-level information. Hence our knowledge that the judgments are generated by two different and conflicting processes will not affect the intuitions generated by each, which will continue to be phenomenologically insistent.^8^

Attention to the same facts can also help to motivate a response to Domsky’s (2005) objections to epistemic reductionism. Domsky argues that the problem of moral luck can arise as insistently in the self-regarding case as in the other-regarding case; since we have direct access to the content of our mental states, the harm cannot serve as evidence for these mental states. Indeed, no harm needs actually to occur: as Domsky also points out, the problem can arise prior to discovering whether our actions have any consequences at all. We need only to consider how things might unfold to have the intuition that if things go badly we are much more blameworthy than otherwise. Again, though, the hypothesis concerns an encapsulated module which generates a nonconceptual output. There is no more reason to think that it is sensitive to information concerning our own mental states than it is to information concerning the mental states of other agents: it produces its characteristic output, given an appropriate stimulus, independent of what other beliefs the person maintains.

## Objections^9^

The account offered here is, admittedly, somewhat speculative. Given that fact, alternative explanations for the pattern of judgments it aims to explain should be taken seriously. One alternative explains moral luck judgments as the consequence of *hindsight bias*. There is extensive evidence that people tend to perceive an event as more predictable or inevitable after they learn that it actually occurred than they would have prior to its occurrence (see Roese and Vohs (2012) for review). It is easy to see why hindsight bias might be thought to have some a role in driving moral luck judgments: the belief that a harmful consequence *has* occurred may be expected to alter perceptions of the moral status of the action via this route.

Hindsight bias directly alters perceptions of the likelihood of an events occurring (eg. Kamin and Rachlinski 1995). By altering perceptions of likelihood it might affect moral judgments, because a change in likelihood entails an alteration in the luckiness of the event. If agent A’s hitting the child when she drives negligently was, in the circumstances, relatively likely, than it is false that A was unlucky to hit the child (probability is directly relevant to the degree to which an event is lucky; see Levy 2011). This would generate an alternative explanation of how knowledge of the consequences of an action affects blameworthiness judgments: if the agent was less the victim of bad luck in causing a harmful consequence, she has less of an excuse. On my hypothesis, perception of the outcome affects our perception of the quality of will of the actor; on the rival, hindsight bias-based alternative, it affects perception of the antecedent likelihood of the event.

As we have already seen, however, Lench et al. (forthcoming) provide evidence that moral luck beliefs are mediated by perceptions of agents’ intentions, which suggests that rather than affecting perceptions of likelihood (alone), knowledge of outcome affects perception of the agent’s state of mind. This suggests in turn that hindsight bias does not explain moral luck judgments by altering perceptions of the likelihood of the harmful consequence.

However, hindsight bias might explain, or partially explain, how perception of the outcome affects perception of mental states. Hindsight bias is known to alter assessments not just of the antecedent probability that an event will occur, but also of how predictable it was. This perception could mediate inferences about the likely state of mind of an agent: if an event was highly predictable, then it might take a worse quality of will to ignore or overlook that fact. If hindsight bias explains inferences about quality of the will, the hypothesis advanced here might simply be a special case of hindsight bias. Several studies have found that hindsight bias influences both the perception of the likelihood of an event and of the negligence of the agent who might have prevented it (LaBine and LaBine 1996; Smith and Greene 2005). Negligence, because it does not require that the agent knew the risks she ran (only that she should have known them) does not directly concern agents’ mental states; nevertheless a judgment of negligence can be interpreted as indirectly a judgment that concerns the quality of an agent’s will. A judgment of recklessness directly concerns mental states, since recklessness requires actual awareness of the risks run. Hastie and Viscusi (1998) and Hastie et al. (2002) found that knowledge of outcome did indeed drive up perceptions of recklessness. These results leave open the possibility that hindsight bias drives moral luck judgments.

Perhaps it is best to distinguish hindsight bias proper, concerning the predictability and antecedent likelihood of an event, from the effect identified here, which concerns the mental states of the actor. We already distinguish three dissociable phenomena under the heading ‘hindsight bias’: memory distortion, inevitability and foreseeability (Roese and Vohs 2012). Foreseeability might explain moral luck judgments, since it might be inferred from the foreseeability of an event that an agent was likely to have foreseen it, or that they must have had a bad quality of will to overlook it. However, the evidence cited by researchers who discuss negligence and recklessness assessments under the heading of hindsight bias is consistent with my alternative hypothesis. Further research dissociating these elements of hindsight bias and disentangling which, if any, drive moral judgments is required to discover whether the hypothesis advanced here should be considered a special case of hindsight bias.

A second objection to the hypothesis advanced here turns on the evidence that judgments of wrongness and permissibility are much more strongly influenced by information about actor’s beliefs and intentions than they are by information about the consequences of their actions (Cushman 2008). My hypothesis, recall, is that attention to the consequences of an action drives up judgments of blameworthiness by altering perceptions of the agent’s mental states. But if attending to the consequences of an action affects perception of the agent’s mental states, and judgments of permissibility and wrongness are responsive to evidence about mental states, then why are these judgments relatively insensitive to information about consequences?

This is a powerful objection. As we have already seen, however, there is evidence (albeit indirect) that blame judgments are mediated by perception of intention (Lench et al. forthcoming). If that’s correct, then we should seek some way to reconcile the evidence that wrongness judgments are relatively insensitive to information about the consequences of actions with the hypothesis that attending to consequences alters perceptions of mental states. These findings are compatible if there is a significant degree of encapsulation of blame judgments. I suggest that blame judgments are driven by a modular process and that the perceptions of agents’ quality of will that mediate these judgments are encapsulated from the (domain general) mechanisms that issue in wrongness judgments.

This is not an ad hoc suggestion. There is evidence that blame judgments are driven by a mechanism that emerges much earlier in development than the mechanism that supports wrongness judgments (see Cushman 2008 for discussion). Mechanisms that develop early are typically mechanisms that require little in the way of effortful processing or domain general information; they are more likely to be domain specific and modular. Such a mechanism may respond to outcomes as cues revelatory of quality of will. In contrast, I suggest, judgments of wrongness and permissibility rely on explicit information concerning agents’ beliefs and desires; the information that Cushman (2008) manipulated.

Cushman (2008) provides support for the claim that blame judgments, unlike wrongness judgments, depend on domain-specific information. Experiments 3 and 4 reported in that paper presented vignettes featuring agents whose beliefs and desires concerning a harmful consequence were systematically varied. In some variations, the consequence occurred by an independent causal route, which had nothing to do with the agent. The surprising finding was that when the harmful consequence occurred by this independent route, participants were much less willing to blame or to punish the agent than when no harmful consequence occurred at all. Cushman calls this ‘blame blocking’; the availability of an alternative target for blame blocks attribution of any blame to the agent, despite her bad quality of will. This kind of counterintuitive blame blocking may indicate the involvement of a mechanism that utilizes domain-specific information, rather than querying the domain-general information available to the person (interestingly, the manipulation did not significantly affect the blame judgments of a minority of participants, which suggests that they overrode the domain-specific mechanism and relied on domain-general information instead. If this hypothesis is correct, we ought to see longer reaction times – indicative of effortful overriding – in this subset of participants). Given the adaptive significance of identifying deliberative defectors, it is unsurprising that mechanisms for detecting a quality of will that predicts defection should be early developing or that they would bypass effortful domain-general cognition.

The objections just considered remain forceful. It would be wrong to suggest that the considerations just offered in response are decisive. Given the support the hypothesis I have offered draws from its capacity to explain a great deal of evidence and from its neat fit with the consensus view on the evolution of punishment, I think it is fair to conclude that it remains a plausible candidate for the best explanation of moral luck judgments.

## Dissolving the Problem of Moral Luck

By explaining why we experience a conflict in intuitions, the account offered here solves the problem of moral luck. It also opens the way to dissolving it. To dissolve the puzzle, in the sense I mean here, is not merely to explain why we experience the conflict in intuitions, but to give us good reasons for preferring one set of intuitions to the other. The account opens the way to a solution by showing us that the intuitions generated by focusing on the consequences of actions are unreliable, when we have sufficiently good independent evidence about agents’ mental states. We are sometimes justified in taking consequences into account in assessing the blameworthiness of agents because consequences are proxies for agents’ mental states. But in moral luck cases, we do not need to utilize such proxies, because we have more direct and more reliable information about the agents’ mental states. In these cases, making use of the proxy tends to cause a less accurate assessment of the actor’s mental states. Since the agents are stipulated to have (relevantly) identical mental states – the same intentions, beliefs, desires, and so on – our disposition to blame one more severely than another is entirely due to the use of an accuracy lowering mechanism. Therefore, when we feel the force of these competing intuitions, we ought to discard those produced by considering the harm caused, in favor of those that are produced by a direct assessment of the actor’s mental states.

Since the mental states of the agents are identical, and our blame judgments should track mental states, each is due the same amount of blame. But how much blame is that? Should they both be judged as severely as the agent who caused the worse harm, or should they be judged only as severely as the agent who was luckier? The answer differs from case to case. Each agent is due an amount of blame that is commensurate with the quality of their will. Here is not the place to develop an account of what quality of will consists in, but it is reasonable to think that in assessing the quality of an agent’s will, one will often or always need to attend, not to the *actual* consequences of their actions, but to their *likely* consequences. When an agent acts recklessly, the fact they knowingly run a risk of a particular magnitude is obviously relevant to the regard they show for morality and for the moral standing of others, and thereby to the quality of their will. Whether we ought to attend to the likely consequences of actions in assessing quality of will in cases in which an agent acts negligently depends on what the right account of negligence is. Some philosophers assimilate these cases to recklessness, arguing that negligence is blameworthy only if the agent knowingly ran the risk of ignorance (e.g., Smith 1983), whereas others believe that agents may be directly responsible in negligence cases (e.g., Clarke 2014). If accounts of the first kind are correct, we need enquire only about the agent’s actual or past mental states to assess their quality of will and can ignore the likely consequences of their actions; if accounts of the second kind are correct, likely consequences may matter to the agent’s quality of will, independently of their beliefs about these consequences.

This account entails that pairs of agents who differ only in the amount of harm their actions cause are due the heavy blame that many are tempted to attribute only to the agent who caused the worse harm when, and only when, that harm was the likely consequence of the action. In cases like that, the agent who caused the less severe harm doesn’t get off more lightly due to their good luck. In other cases, though, the less severe blame is due to both agents: when the less severe harm is more likely. In cases like that, the agent who caused the more serious harm has their blame moderated, by attention to the fact that they were unlucky. Sometimes the agents are due an amount of blame somewhere between the amount we might be disposed to attribute to the first and to the second: when both are unlucky (the first in causing a harm much worse than he had any right to fear and the second in causing a result less serious than he had any right to expect). In other words, in assessing the amount of blame due to agents, we need to control for luck.^10^ In no case will we need to attend to the *actual* consequences. It is the likely consequences that matter (of course, the actual consequences will be identical to the likely consequences in most cases).

Evolution has disposed us, for good reasons, to be implicitly committed to a theory of blame according to which blame ought to be commensurate to actors’ mental states, and also to be disposed to attribute worse mental states to agents who cause worse harms than to those whose actions have less bad consequences. Sometimes, these implicit commitments conflict. When they do, we ought to reject the intuitions produced by attention to the actual consequences of actions, because these intuitions are produced by a mechanism that is unreliable. The puzzle of resultant moral luck is thereby solved and dissolved.^11^

## Footnotes

1 It is worth noting that there is evidence that the conflict in intuitions reported here is not confined to philosophers. Lench et al. (forthcoming) report three studies which measured participants’ explicit beliefs about moral luck as well as their judgments concerning the blameworthiness and liability to punishment of the agents who feature in moral luck cases. They found that a majority of subjects report an explicit rejection of the claim that blameworthiness or liability to punishment can be affected by moral luck but that many are nevertheless disposed to blame and punish an agent who unluckily produces harm more severely than an otherwise identical, but luckier, agent. I thank an anonymous referee for drawing my attention to this paper.

2 Epistemic accounts of moral luck have been criticized on the grounds that they unjustifiably overgeneralize a solution which at best only explains some cases of moral luck (Concepcion 2002; Schinkel 2009). This criticism may well be justified, but it does not apply to the account offered here which is intended only to apply to resultant moral luck. It certainly cannot be generalized to what Nagel (1979) calls constitutive luck, since the solution turns on the claim that our judgments concerning agents’ mental states may be unreliable, whereas constitutive luck is an agent’s luck in (*inter alia*) what mental states she actually has.

3 A similar puzzle may arise with regard to praise: intuitions about actions that are praiseworthy seem weaker, so it is hard to be sure. The solution I offer here can easily be generalized to cover praise as well as blame.

4 It is, admittedly, very hard to disentangle the sense of blame in the basic desert sense from other senses that are not at issue here, but there are grounds for thinking that the kind of blame for which the appropriateness of what Williams (1981) calls agent regret – regret that one has been the cause of some significant harm – is sometimes taken as evidence is not blame in this sense. Wolf (2001) claims that taking responsibility for the consequences of one’s actions by evincing agent regret is the expression of a ‘nameless virtue’. That seems to me to be exactly right; self-blaming, of the kind she thinks is required, is justified (if it is justified at all) by considerations concerning the kind of character traits we should admire and want to cultivate, not by considerations concerning what we deserve. I thank a referee for this journal for pressing me on this issue.

5 This way of talking is not intended to express a commitment to the controversial claim that selection processes operate on groups, as well as genes or individuals. The ‘group’ talk here can be translated into talk of individual fitness or even gene frequencies.

6 Further independent evidence for this claim comes from neuroimaging work that shows that subjects judging morally significant actions engage in spontaneous mental inferences when they lack explicit information about actors’ beliefs and desires; see Young and Tsoi (2013) for review.

7 The overall pattern of judgments might be made consistent if the blame responses were justified on different bases: a disposition to blame on the basis of harm justified by considerations of deterrence and a disposition to blame on the basis of mental states justified by retributivist considerations. I know of no direct empirical evidence against this suggestion, but there is indirect evidence against it. First there is the evidence reviewed in Carlsmith and Darley (2008), indicating that blame and punishment judgments exhibit very little sensitivity to considerations of deterrence. Second, the suggested explanation seems to predict that we will not experience any conflict when we contemplate resultant moral luck scenarios: rather, we will be comfortable in judging that the agent who causes the worse harm should be punished more severely than his luckier counterpart.

8 Lench et al. (forthcoming) found that considering counterfactuals – how the scenario featuring moral luck might have ended – made a difference to subjects’ blame and punishment judgments, bringing them more into line with their explicit luck beliefs by having them more closely track actors’ intentions (though this effect was observed only when subjects considered how events might have gone worse, not when they considered how they might have gone better). As a referee for this journal emphasizes, this result indicates that moral luck judgments are not entirely resistant to correction. This evidence is consistent with my claim about recalcitrance, which concerns the intuition generated by considering the harms caused by an action; on my hypothesis considering the counterfactual generates a new and stronger intuition. It is also consistent with my account that considering counterfactuals in which things went better did not alter judgments: the mechanism may generate intuitions which correspond in strength to the severity of the harm caused, and the strongest intuition may be the only salient one.

9 Both objections discussed in this section – the suggestion that the hypothesis might be generated by hindsight bias and that it might be inconsistent with evidence concerning judgments of wrongness and permissibility – were raised by an anonymous referee for this journal.

10 Controlling for luck requires a metric for measuring it. I have sketched such a metric elsewhere (Levy 2011).

11 I am very grateful to two anonymous referees for the *Review of Philosophy and Psychology* for very helpful and very challenging comments. An earlier version of this paper was presented at a meeting of the Agency and Moral Cognition Network, at Macquarie University, Sydney, and at a work in progress seminar at the University of Oxford. I am grateful to the audience members at both venues for helpful feedback. I am also grateful to the Australian Research Council for supporting my research by generous provision of a Future Fellowship.

## References

[CR1] Adler JE (1987). Luckless desert is different desert. Mind.

[CR2] Alicke MD (2000). Culpable control and the psychology of blame. Psychological Bulletin.

[CR3] Boyd R, Gintis H, Bowles S, Richerson PJ (2003). The evolution of altruistic punishment. Proceedings of the National Academy of Science.

[CR4] Carlsmith KM, Darley JM (2008). Psychological aspects of retributive justice. Advances in Experimental Social Psychology.

[CR5] Clarke R, Mele AR (2014). Negligent action and unwitting omission. Surrounding free will.

[CR6] Concepcion DW (2002). Moral luck, control, and the bases of desert. Journal of Value Inquiry.

[CR7] Cushman FA (2008). Crime and punishment: differential reliance on causal and intentional information for different classes of moral judgment. Cognition.

[CR8] Cushman FA, Dreber A, Wang Y, Costa J (2009). Accidental outcomes guide punishment in a ‘trembling hand’ game. PloS One.

[CR9] Cushman F, Kim S, Richard J, Brett C, Ben F (2013). The role of learning in punishment, prosociality, and human uniqueness. Cooperation and its evolution.

[CR10] Domsky D (2005). Tossing the rotten thing out: eliminating bad reasons not to solve the problem of luck. Philosophy.

[CR11] Fodor J (1983). The modularity of mind: an essay on faculty psychology.

[CR12] Frey UJ, Rusch H (2012). An evolutionary perspective on the long-term efficiency of costly punishment. Biology and Philosophy.

[CR13] Hastie R, Viscusi WK (1998). What juries can’t do well: the jury’s performance as a risk manager. Arizona Law Review.

[CR14] Hastie R, Schkade DA, Payne JW, Cass S, Reid H, Payne JW, Kip WV (2002). Looking backward in punitive judgments: 20–20 vision?. Punitive damages: how juries decide.

[CR15] Kamin KA, Rachlinski JJ (1995). Ex post ≠ ex ante: determining liability in hindsight. Law and Human Behavior.

[CR16] LaBine SJ, LaBine G (1996). Determinations of negligence and the hindsight bias. Law and Human Behavior.

[CR17] Lench, Heather C., Domsky, Darren, Smallman, Rachel & Darbor, Kathleen E. (Forthcoming) ‘Beliefs in moral luck: When and why blames hinges on luck’. *British Journal of Psychology* DOI: 10.1111/bjop.12072. 10.1111/bjop.12072 24738953

[CR18] Levy N (2011). Hard luck: how luck undermines free will and moral responsibility.

[CR19] McKenna M (2012). Conversation and responsibility.

[CR20] Nagel T (1979). Moral luck’. In mortal questions.

[CR21] Nakao H, Machery E (2012). The evolution of punishment. Biology and Philosophy.

[CR22] Richards N (1986). Luck and desert. Mind.

[CR23] Roese NJ, Vohs KD (2012). Hindsight bias. Perspectives on Psychological Science.

[CR24] Schinkel A (2009). The problem of moral luck: an argument against its epistemic reduction. Ethical Theory and Moral Practice.

[CR25] Smith AC, Greene E (2005). Conduct and its consequences: attempts at debiasing jury judgments. Law and Human Behavior.

[CR26] Smith H (1983). Culpable ignorance. Philosophical Review.

[CR27] Sterelny K (2012). The evolved apprentice: how evolution made humans unique.

[CR28] Thomson JJ (1989). Morality and bad luck. Metaphilosophy.

[CR29] Williams B (1981). Moral luck’. In moral luck.

[CR30] Wolf S (2001). The moral of moral luck. Philosophic Exchange.

[CR31] Young L, Tsoi L (2013). When mental states matter, when they don’t, and what that means for morality. Social and Personality Psychology Compass.

